# Halogenated quinoline kill agent rapidly induces iron starvation of *Staphylococcal* biofilms

**DOI:** 10.1007/s00044-025-03471-9

**Published:** 2025-09-16

**Authors:** Robert W. Huigens, Ke Liu, Nana Shao, Qiwen Gao

**Affiliations:** 1 Department of Pharmaceutical and Biomedical Sciences, College of Pharmacy, University of Georgia, Athens, GA, USA; 2 Department of Chemistry, Franklin College of Arts and Sciences, University of Georgia, Athens, GA, USA; 3 Department of Infectious Diseases, College of Veterinary Medicine, University of Georgia, Athens, GA, USA; 4 Department of Medicinal Chemistry, Center for Natural Products, Drug Discovery and Development (CNPD3), College of Pharmacy, University of Florida, Gainesville, FL, USA

**Keywords:** Bacterial biofilms, Biofilm eradication, Halogenated quinoline, Iron starvation

## Abstract

Bacterial biofilms are surface-attached communities comprised of slow- or non-replicating bacteria. Transcriptomic responses of bacterial biofilms to anti-biofilm small molecules have been largely unexplored, with existing studies typically involving long treatment periods (>18 h). In this study, we used a halogenated quinoline biofilm-killing agent (RA-HQ-12) to investigate the transcriptional responses of MRSA and *S. epidermidis* biofilms. Utilizing RT-qPCR, we observed RA-HQ-12 activated iron uptake pathways in both MRSA and *S. epidermidis* biofilms after 4 h treatment at 1 μM. A time-course analysis further revealed dynamic variation in up- and down-regulation patterns of various target genes (*sbnC, isdB, opp1C, ribA, nasE, and crtM*), shedding light on the time-dependent dynamics of biofilm responses to RA-HQ-12.

## Graphical Abstract

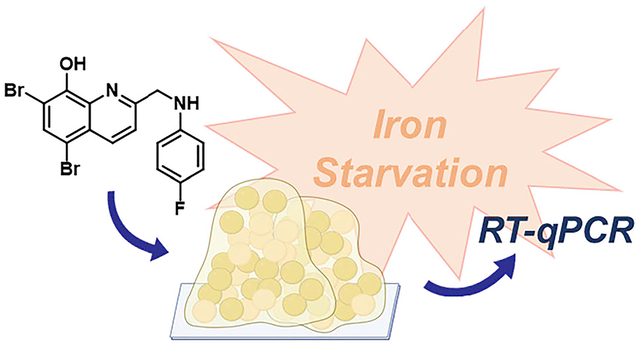

## Introduction

Bacteria exhibit two distinct lifestyles that include the planktonic state, where bacteria exist as rapidly-dividing individual cells, and the biofilm state, where communities of slow- and non-replicating bacteria form aggregates on surfaces [1–4]. It is important to distinguish planktonic from biofilm-associated bacteria as each class of our antibiotic arsenal was initially discovered as growth inhibitors that target actively-replicating planktonic cells [3, 5]. In contrast, biofilms are comprised of metabolically-dormant “persister” cells that are notorious for their innate tolerance to all known classes of antibiotics [2, 3, 6]. Biofilms persist and thrive in hostile environments and their adaptive features make them particularly challenging to treat as they are credited as the primary cause of chronic and recurring infections [2, 7, 8]. Biofilms are a major cause of human infections, with nearly all indwelling-related infections being linked to biofilm formation [1, 9]. It is estimated that biofilms account for over 65% of nosocomial infections, around 80% of chronic infections, and 60% of all bacterial infections in humans [10–12]. Given the significant burden biofilm infections place on healthcare systems [13–15], there is an urgent need to develop novel therapeutic strategies to address the many problems associated with biofilms in clinical settings.

**Fig. 1:**
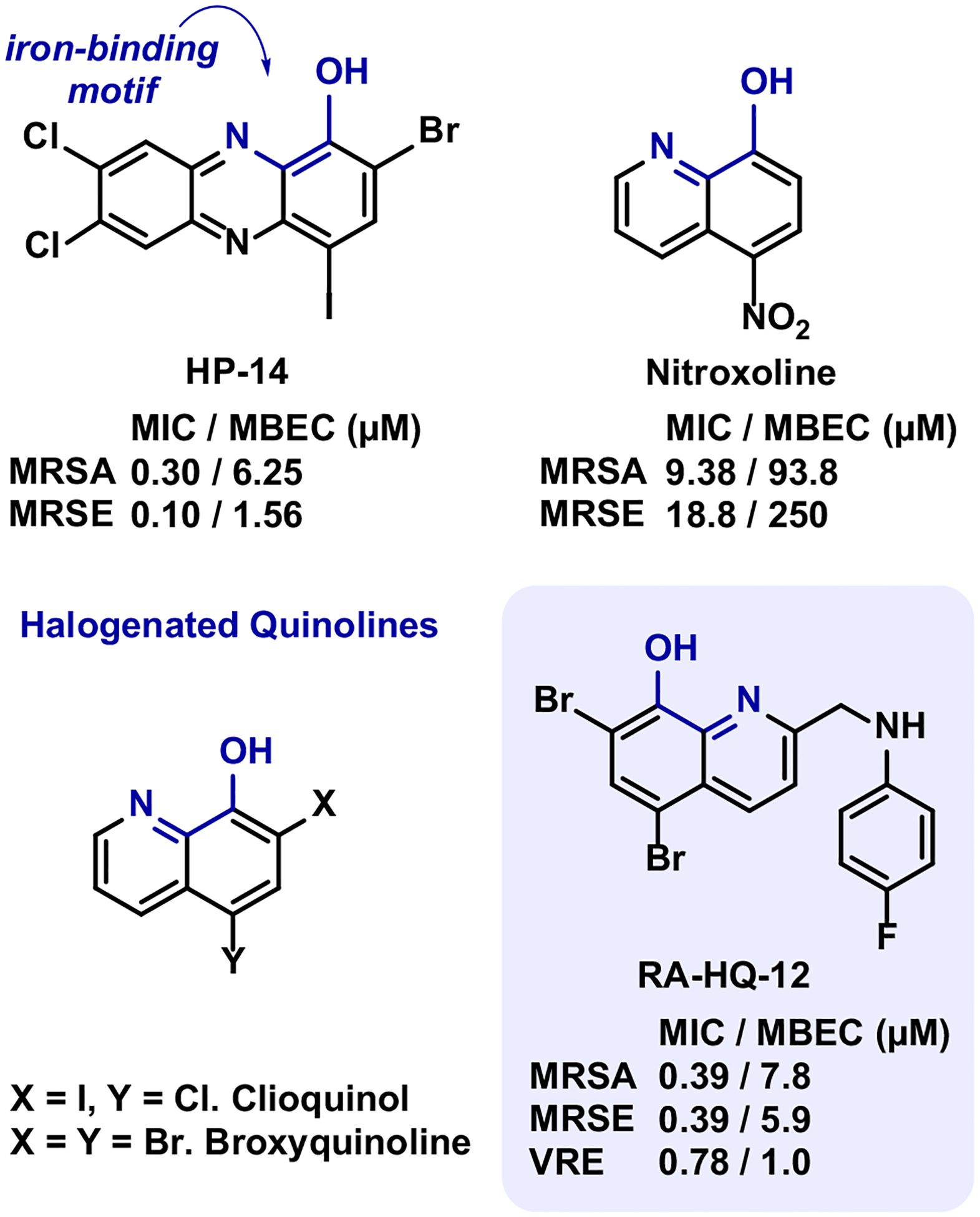
Chemical structures of halogenated phenazine analogue HP-14, nitroxoline, clioquinol, broxyquinoline and RA-HQ-12. This collection of compounds displays an array of antibacterial activities with HP-14 and RA-HQ-12 demonstrating potent biofilm-eradication activities

Small molecules that can operate through mechanisms that target and kill both free-floating planktonic bacteria and surface-attached biofilms represent promising therapeutic candidates for treating persistent biofilm-associated infections [3, 5]. In previous work, we found 2,4-dibromo-1-hydroxyphenazine (HP-1) to exhibit potent antibacterial activity against *Staphylococcus aureus* and *Staphylococcus epidermidis*, with minimum inhibitory concentrations (MICs) ranging from 0.78–1.56 μM [16]. In later work, we found several halogenated phenazine analogues, including HP-14 and nitroxoline, to eradicate (kill) MRSA biofilms through the rapid induction of iron starvation in transcript profiling experiments (Fig. 1) [8, 17–19].

Our efforts related to the eradication of bacterial biofilms have expanded from halogenated phenazines to novel halogenated quinolines (HQ) through scaffold hopping (Fig. 1) [20–22]. Broxyquinoline and clioquinol are known 8-hydroxyquinolines that have demonstrated numerous pharmacological properties often associated with modes of action related to their ability to chelate metal cations (e.g. topical antiseptic, intestinal amebicide, neurological disorders including Alzheimer’s disease, antibacterial) [23–29]. Interestingly, clioquinol has been reported to inhibit biofilm formation in *Escherichia coli* through the disruption of divalent cation homeostasis; however, eradication (killing) of biofilms was not assessed in these studies [30]. Our lab showed that biofilm-killing HQs could be developed through synthetic modifications of the 2-position of the quinoline nucleus [21, 22], which lead to the identification of RA-HQ-12 that eradicates methicillin-resistant *S. aureus* (MRSA, minimum biofilm eradication concentration or MBEC = 7.8–93.8 μM), methicillin-resistant *S. epidermidis* (MRSE, MBEC = 5.9 μM), and vancomycinresistant *Enterococcus faecium* (VRE, MBEC = 1.0 μM) biofilms with outstanding potency in Calgary Biofilm Device (CBD) assays without lysing human red blood cells at 200 μM [22].

In this study, we used RA-HQ-12 as a probe to investigate the transcriptional responses of *Staphylococcal* biofilms to better understand select target gene expression profiles related to biofilm viability. Reverse transcription quantitative real time polymerase chain reaction (RT-qPCR) analysis revealed that RA-HQ-12 activated iron uptake pathways in MRSA and *S. epidermidis* biofilms after 4 h of treatment. A time-course analysis showed dynamic changes in the up- and down-regulation of key genes involved in MRSA biofilms response to RA-HQ-12 treatment following 2, 8 and 20 h.

## Results and discussion

Our experimental design to probe the transcriptional impacts RA-HQ-12 has on select genes in *Staphylococcal* biofilms was based on previous findings from work with HP-14 and nitroxoline [8, 17]. We reported RA-HQ-12 to kill MRSA-1707 biofilms with a minimum biofilm eradication concentration (MBEC) of 7.8 μM in Calgary Biofilm Device assays (where biofilms were treated with RA-HQ-12 for 24 h) [22]; therefore, we used this MBEC value to select a test concentration to investigate transcriptional responses in this work. Initial experiments with MRSA-1707 biofilms focused on 8 target genes following RA-HQ-12 treatment at 1 μM (1/8 MBEC) for 4 h as we aimed to define initial transcriptional changes.

**Fig. 2:**
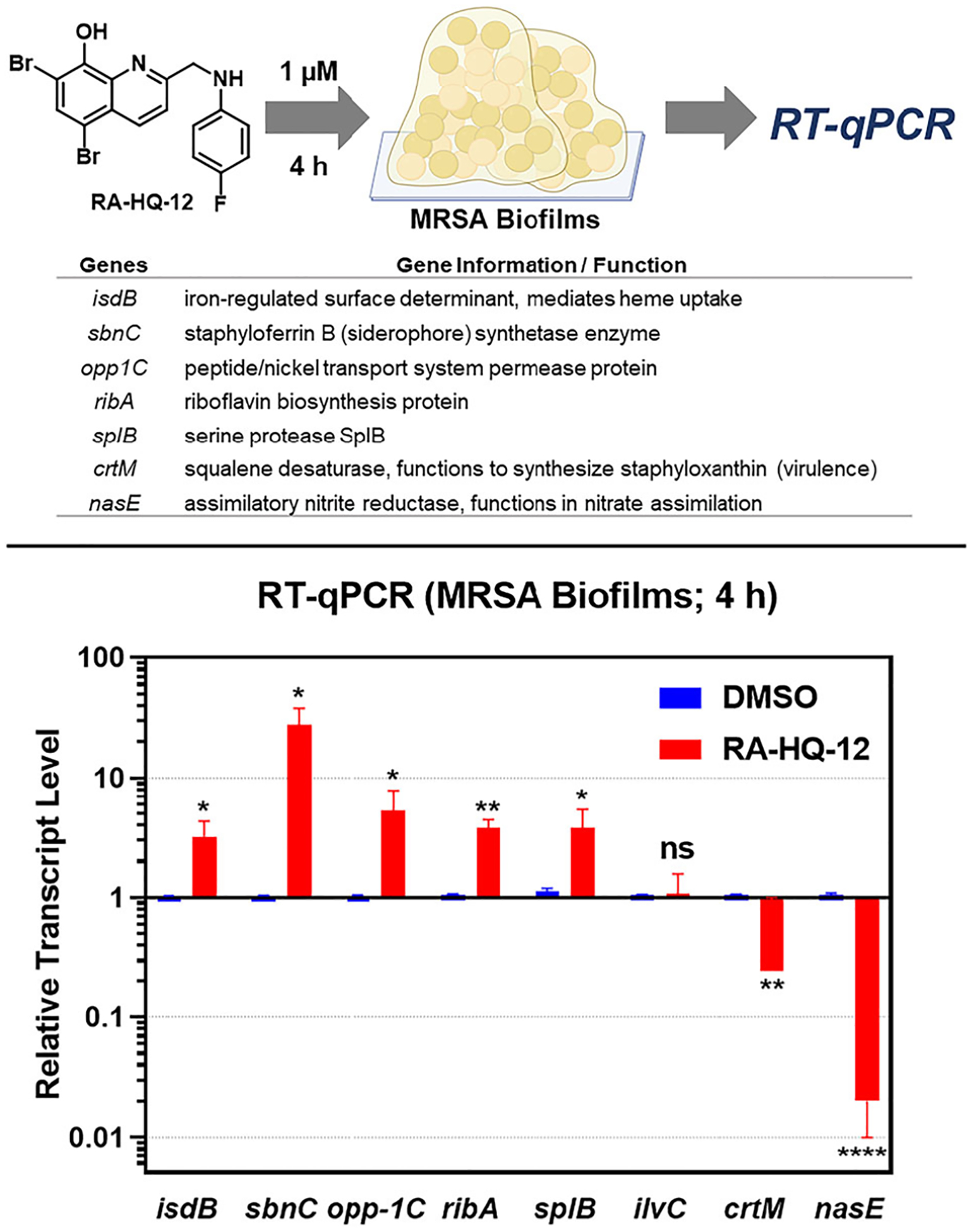
Initial RT-qPCR experiments to probe select genes in MRSA 1707 biofilms treated by RA-HQ-12 at 1 μM for 4 h. *p value ≤ 0.05, **p value ≤ 0.01, ****p value ≤ 0.0001 (Student’s t-test). Error bars were omitted when they were smaller than the data column. ns = not significant (change)

Using RT-qPCR, we analyzed MRSA-1707 biofilms treated with RA-HQ-12 (at 1 μM) and vehicle (DMSO) alone for 4 h in initial experiments (Fig. 2). We were curious to probe transcript levels of select iron starvation biomarkers (*isdB, sbnC*) [31–35] correlated with the biofilm-eradicating properties of RA-HQ-12 as we found this compound to directly bind iron(II) in UV-vis experiments (see Supporting Information). Iron uptake is critical for nearly all bacteria to survive and they have multiple mechanisms to obtain iron as this essential element is unable to diffuse through bacterial membranes [36–38]. The iron uptake biomarkers for this work include: (1) *isdB*, which is the iron-regulated surface determinant B involved in iron acquisition from heme uptake [31, 32], and (2) *sbnC*, the staphyloferrin B synthetase gene involved in siderophore biosynthesis to uptake iron from the environment [33–35]. Initial RT-qPCR experiments show RA-HQ-12 significantly increased transcription levels of *isdB* (3.2 ± 0.9-fold upregulation) and *sbnC* (27.7 ± 8.3-fold upregulation) in MRSA biofilms following 4 h treatment, suggesting this compound induces rapid iron starvation.

In addition to iron starvation biomarkers, we inspected other target genes in MRSA biofilm potentially impacted by RA-HQ-12 treatment in the initial 4 h treatment experiments based on previous work [8, 17] (Fig. 2), including: oligopeptide transport gene *opp-1C* (encoding a peptide/nickel transport system permease protein) [39], *ribA* (riboflavin biosynthesis protein) [40], *splB* (serine protease) [41], ilvC (ketol-acid reductoisomerase, biosynthesis of branched-chain amino acids) [42], *crtM* (squalene desaturase; functions to synthesize staphyloxanthin) [43], *nasE* (assimilatory nitrite reductase, involved in nitrate assimilation) [44]. We found RA-HQ-12 to upregulate the transcription of several genes in MRSA biofilms following 4 h treatment, including: *opp-1C* (5.3-fold upregulation), *ribA* (3.8-fold), and *splB* (3.8-fold). In addition, RA-HQ-12 downregulated the following two genes in MRSA biofilms after the 4-h treatment: *crtM* (4.1-fold reduction), and *nasE* (50-fold). Finally, we found RA-HQ-12 to have no effect on the transcription of *ilvC*.

**Fig. 3:**
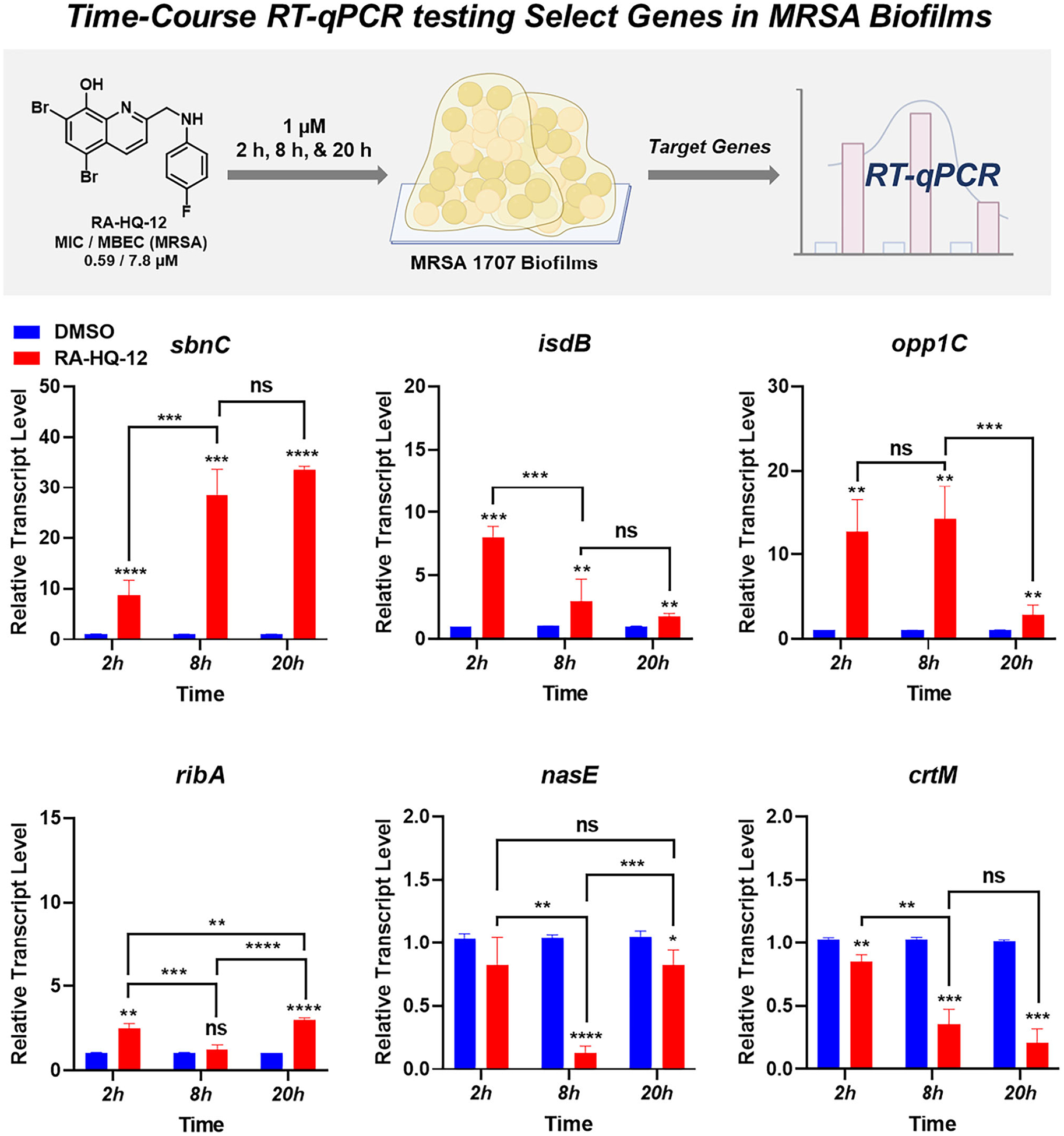
Time-course RT-qPCR experiments evaluating the impacts RA-HQ-12 has on select genes in MRSA biofilms. *p value ≤ 0.05, **p value ≤ 0.01, ***p value ≤ 0.001, ****p value ≤ 0.0001 (Student’s t-test). The error bars were omitted when they were smaller than the data column. ns = not significant (change)

Following the 4-h treatment of MRSA-1707 biofilms with RA-HQ-12, we performed a time-course analysis of 6 gene transcripts that were found to be up- or downregulated during initial experiments (Fig. 3). Here, we designed time-course studies to probe transcript profiles in MRSA biofilm target genes after 2, 8, and 20 h of RA-HQ-12 treatment (at 1 μM) from biofilms originating from the same overnight cultures to obtain the most ideal time point analysis. Interestingly, despite the focused number of gene transcripts analyzed in these experiments, we found select transcripts that increased expression over time, decreased expression over time, or demonstrated fluctuating expression levels over the experimental time course.

**Fig. 4:**
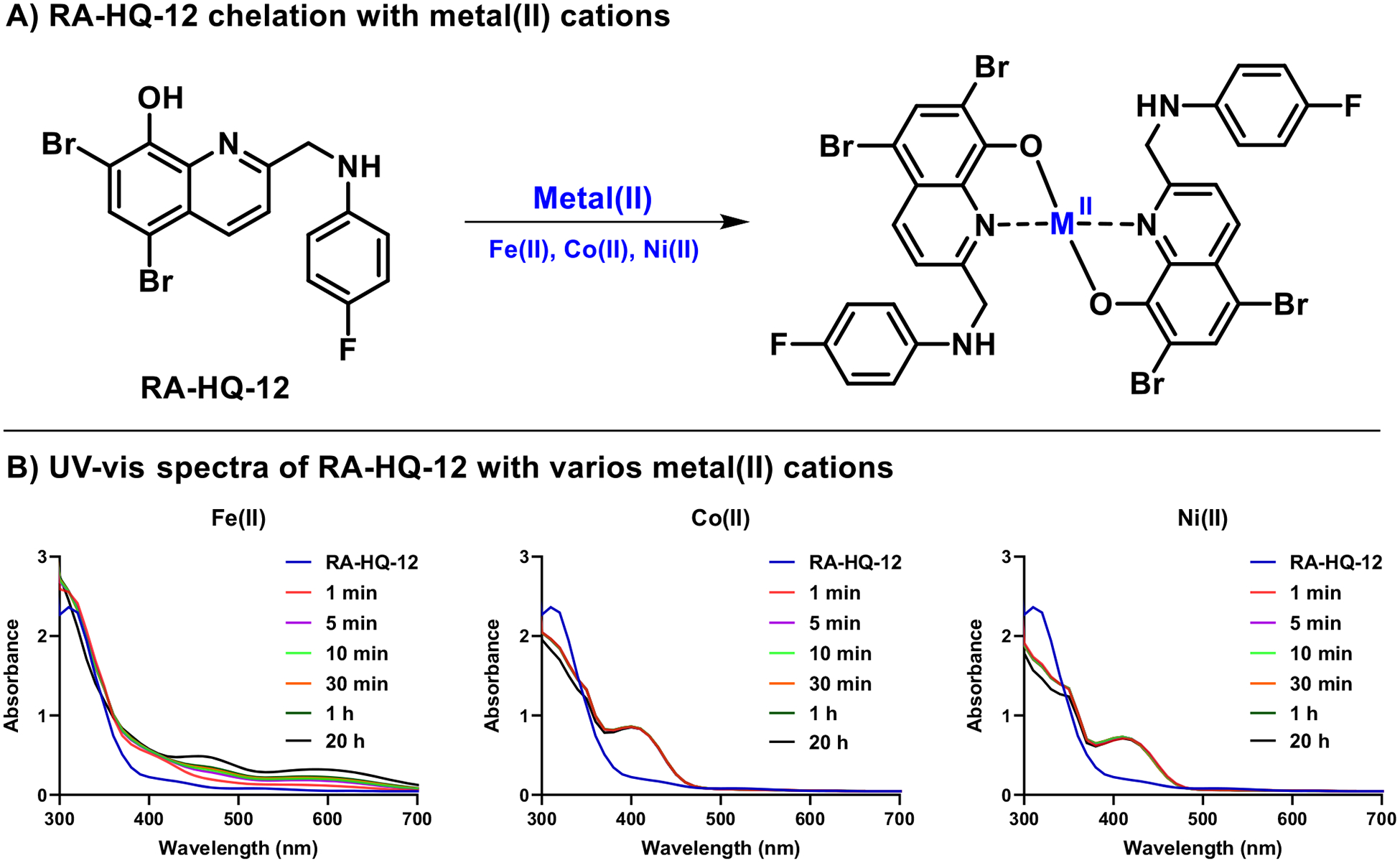
UV-vis spectroscopy shows RA-HQ-12 directly binds metal (II) cations. **A** RA-HQ-12 was used in a 2:1 ratio to form chelates with iron(II), cobalt(II), and nickel(II). **B** UV-vis spectroscopy was used to observe RA-HQ-12 binding metal(II) cations with elevated absorbances observed at ~380–480 nm for nickel(II)/cobalt(II) and ~400–700 nm for iron(II). Interestingly, RA-HQ-12 rapidly binds Co(II) and Ni(II) while chelation to iron(II) was found to be slower under these experimental conditions

The time course experiments in MRSA biofilms showed increased expression levels of *sbnC* (staphyloferrin B synthetase gene involved in siderophore biosynthesis) over the selected time course parameters with an upregulation of 8.7-fold after 2 h, 28.4-fold after 8 h, and 33.5-fold after 20 h following RA-HQ-12 treatment (Fig. 3). Staphyloferrin B is a citrate-based polycarboxylate siderophore utilized by *S. aureus* to bind iron(III) from the surrounding environment and facilitate uptake/utilization for critical processes (e.g., enzyme functions related to respiration, electron transport, amino acid synthesis; virulence) [33, 45]. Interestingly, while RA-HQ-12 treatment led to increases in *sbnC* expression over the time course, this small molecule led to an initial upregulation of *isdB* (iron-regulated surface determinant B involved in iron acquisition from heme) after the 2 h treatment, but then decreased in 8 and 20 h time points. Specifically, RA-HQ-12 treatment of MRSA biofilms led to an initial 8.0-fold upregulation of *isdB* after 2 h, which then decreased to a 3.7-fold upregulation after 8 h, and 1.8-fold upregulation after 20 h. It is interesting to note the contrasting expression profiles for each of these iron uptake mechanisms (biomarkers) in MRSA biofilms and we feel further exploration with additional iron uptake systems in *S. aureus* would be of incredible value as these pathways have been an area of interest to the development of next-generation antibiotics [46, 47]. We used UV-vis spectroscopy to show that RA-HQ-12 directly binds iron(II) cations and believe this molecule induces iron starvation as a result of direct chelation of this critical element for *S. aureus* biofilms to thrive (Fig. 4).

Similar to the initial 4-h experiment in MRSA-1707 biofilms, RA-HQ-12 significantly upregulated *opp1C* expression 12.7-fold after 2 h and 14.2-fold after 8 h in the time course experiments (Fig. 3); however, a significant decline was observed for *opp1C* expression following the 20-h treatment which showed a moderate 2.9-fold upregulation. It is interesting to note that the S. *aureus* Opp1 ABC transporter has cobalt and nickel transporter activity [39] and points to the possibility of HQs chelating these trace metals leading to the disruption of critical biological processes (e.g., enzyme function, virulence) [48–50]. As a quick follow up to our findings with *opp1C*, we showed that RA-HQ-12 rapidly binds cobalt(II) and nickel(II) cations using UV-vis spectroscopy (Fig. 4). RA-HQ-12 also upregulated *ribA* expression at 2 h (2.5-fold) and 20 h (3.0-fold); however, we did not observe transcriptional changes of *ribA* following 8 h of RA-HQ-12 treatment. Riboflavin plays a key role in various crucial cellular functions, including involvement of redox reactions critical for energy metabolism [40]. Bacteria are known to utilize regulatory processes where iron levels influence the expression of riboflavin biosynthesis and uptake systems [51]. Riboflavin increases iron bioavailability and improves iron acquisition pathways in bacteria.

RA-HQ-12 downregulated *nasE*, and *crtM* in time course experiments in MRSA-1707 biofilms (Fig. 3). Interestingly, *nasE* was not significantly downregulated following 2 h, then showed a 7.7-fold reduction in expression after 8 h, and finally showed minimal (1.3-fold) downregulation after 20 h treatment of RA-HQ-12. *S. aureus* utilizes assimilatory nitrate reductase (*nas*) enzymes, which contain the ironassociated co-factor siroheme, to reduce nitrate to ammonia [44, 52]. Additionally, RA-HQ-12 treatment led to minimal reductions in *crtM* expression after 2 h, then induced a 2.9-fold downregulation after 8 h, and a 4.8-fold down-regulation after 20 h. Carotenoid (crt) biosynthesis is critical for the production of staphyloxanthin, the pigment that gives *S. aureus* the characteristic golden color and virulent factor [43, 53]. Staphyloxanthin possesses antioxidant properties that are believed to evade oxidative stress-related host immune responses during infection (e.g., hydrogen peroxide, superoxide radical, and neutrophil killing) [54, 55]. Inhibitors of staphyloxanthin production are being pursued to develop antivirulence therapies against *S. aureus* [56]. In the context of these studies, we believe the delayed downregulation of *nasE* and *crtM* are indicative of secondary responses to the iron starvation RA-HQ-12 inflicts on MRSA biofilms.

**Fig. 5:**
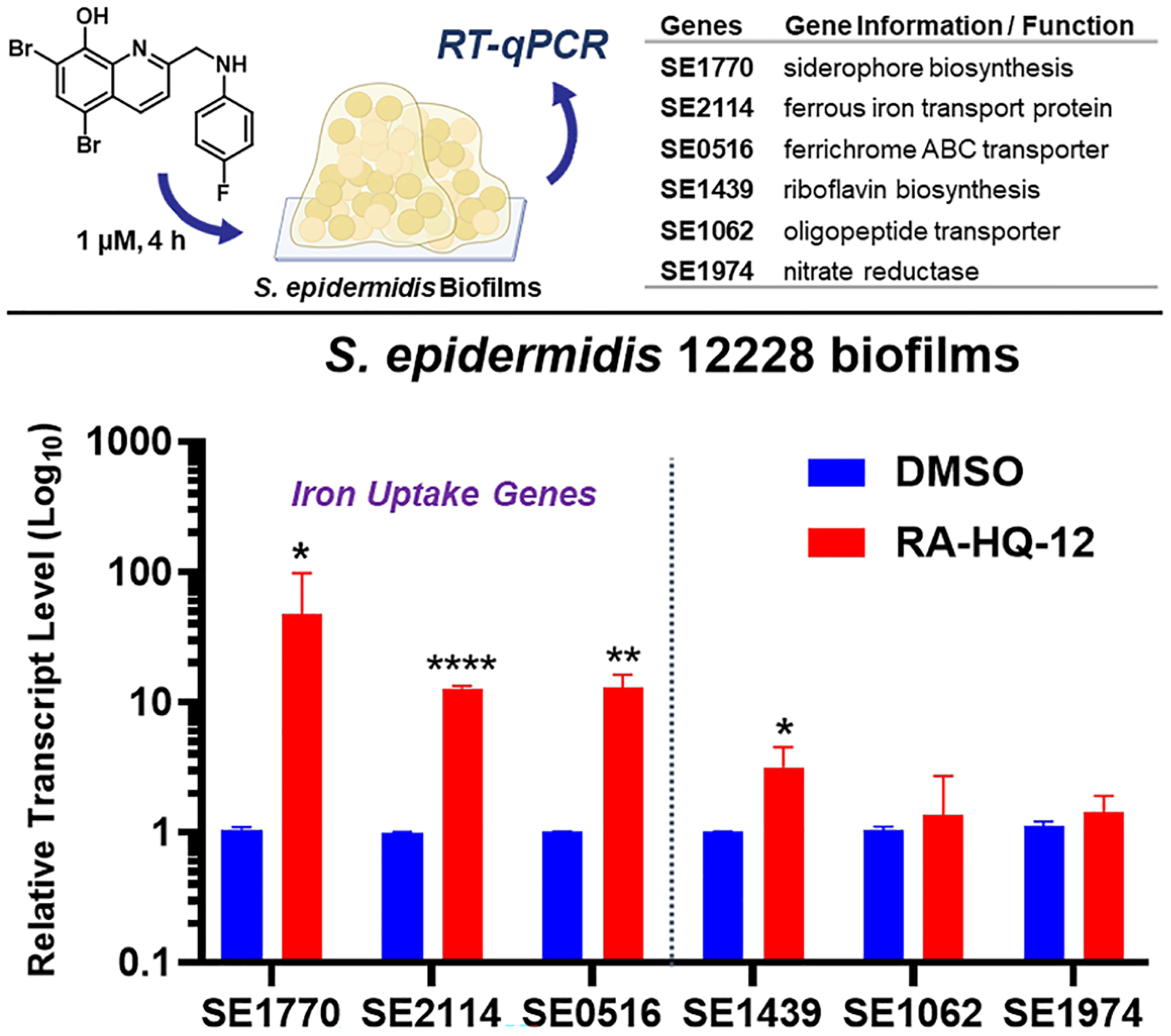
RT-qPCR experimental findings to determine the impacts RA-HQ-12 has on select gene transcripts in S. epidermidis 12228 biofilms after 4 h. *p value ≤ 0.05, **p value ≤ 0.01, ****p value ≤ 0.0001 (Student’s t-test)

We then tested RA-HQ-12 in *S. epidermidis* biofilms to evaluate target genes analogous to those selected in our MRSA experiments (Fig. 5). *S. epidermidis* has emerged as a leading cause of implant-associated infections, due to their ability to form robust biofilms [57]. *S. epidermidis* biofilms utilizes siderophore-mediated acquisition mechanisms to obtain iron [57–59]; therefore, we were interested to evaluate RA-HQ-12’s impact on iron uptake pathways of this pathogen. Following a 4-h treatment with RA-HQ-12 at 1 μM, we found multiple iron uptake biomarkers significantly upregulated in *S. epidermidis* 12228 biofilms, including: SE1770 (siderophore biosynthesis; 47.8-fold upregulation), SE2114 (ferrous iron transport protein; 12.8-fold), and SE0516 (ferrichrome ABC transporter; 13.1-fold). In addition to the iron acquisition transcripts, we observed upregulation of riboflavin biosynthesis gene SE1439 (3.2-fold) following RA-HQ-12 treatment; however, oligopeptide transporter gene SE1062 and nitrate reductase-encoding gene SE1974 transcripts were not altered in these experiments. From these experiments, RA-HQ-12 demonstrates rapid iron starvation in *S. epidermidis* 12228 biofilms as its primary mode of eradication.

## Conclusions

In conclusion, RA-HQ-12 was used to probe focused transcriptional responses in MRSA and *S. epidermidis* biofilms to better understand its mode of action and biofilm viability. Using an orchestrated series of RT-qPCR experiments, we showed RA-HQ-12 rapidly activates multiple iron uptake pathways in MRSA and *S. epidermidis* biofilms following 4 h treatment at sub-MBEC concentrations (1 μM). A detailed time-course analysis of MRSA biofilms treated with RA-HQ-12 further revealed dynamic variation in up- and downregulation patterns of target genes of interest (*sbnC, isdB, opp1C, ribA, nasE, crtM*) following 2-, 8-and 20-h treatments shedding light on time-dependent biofilm responses to this kill agent. These collective findings with RA-HQ-12 are indeed critical as our arsenal of conventional antibiotic therapies are unable to eradicate persistent biofilms that are the primary factor in chronic and recurring bacterial infections.

## Experimental section

### General

The methicillin-resistant *Staphylococcus aureus* ATCC BAA-1707 (MRSA 1707, or *S. aureus* MW2) and *Staphylococcus epidermidis* ATCC 12228 were obtained from the American Type Culture Collection in Manassas, VA, USA. The bacterial cultures were grown in tryptic soy broth with 0.5% glucose (TSBG). The cultures were incubated in a shaker incubator at 37 °C at 260 rpm. Growth was monitored via optical density at 600 nm (OD_600_) with a spectrophotometer. RA-HQ-12 was stored as dimethyl sulfoxide (DMSO) stocks at room temperature and shielded from light. To preserve the integrity of the DMSO stock solutions, freeze-thaw cycles were avoided.

### Total RNA extraction from biofilms

*Biofilm Formation*. MRSA 1707 or *S. epidermidis* 12228 was cultured in fresh tryptic soy broth supplemented with 0.5% glucose (TSBG) and incubated at 37 °C until the optical density at 600 nm (OD_600_) reached 0.8–1.0. Then, 1 mL of inoculated culture was transferred to a 24-well plate precoated with 0.1% gelatin. The plate was then incubated for 20 h at 37 °C under static conditions to allow for biofilms to form. Following this incubation period, the contents of the 24-well plates were discarded leaving only the biofilms that adhered to the bottom of the wells. *Treating Established Biofilms with* RA-HQ-12. RA-HQ-12 was added into established biofilms of MRSA 1707 or S. *epidermidis* 12228 in TSBG at a concentration of 1 μM. As a negative control, an equal volume of DMSO (vehicle) was added. The plate was then incubated under static conditions at 37 °C for 20 h. Following this incubation period, the liquid culture was discarded leaving only the surface-attached biofilms. *Total RNA Extraction from Bacterial Biofilms*. The biofilm was resuspended in 0.5 mL RNA-protect Bacteria Reagent (Qiagen) and transferred into 2 mL tubes. The bacterial cells were centrifuged for 1 min at 15,000 × g, and the supernatant was then discarded. Total RNA was extracted using the RiboPure RNA Purification Kit, Bacteria (Invitrogen, cat # AM1925), following the manufacturer’s instructions. Genomic DNA was digested using the materials provided in the kit. RNA *Quality Control*. RNA concentration was determined on Qubit^®^ 2.0 Fluorometer (ThermoFisher/Invitrogen, Grand Island, NY), and RNA quality was assessed using the Agilent 2100 Bioanalyzer (Agilent Technologies, Inc.). Total RNA with RNA integrity numbers (RIN) ≥ 6.8 were used for quantitative real-time PCR. All data obtained were generated from three independent experiments. These experiments were performed using the same experimental protocol published by our lab in previous work [8, 17].

### Quantitative real-time PCR (RT-qPCR)

Total RNA was isolated from MRSA 1707 or S. *epidermidis* 12228 biofilms treated with RA-HQ-12 or vehicle (DMSO) alone. Real-time PCR reactions were conducted using the Power SYBR Green RNA-to-CT 1-Step Kit (Applied Biosystems, cat# 4389986) using the manufacturer’s guidelines. The primers used during the courses of these studies are detailed in the Supporting Information associated with this manuscript. The housekeeping gene *ptaA* was used as the reference gene. For PCR experiments, all contents (see table below for details) were added to 1.5 mL Eppendorf tubes, mixed by centrifugation at 10,000 × g for 1 min, and 20 μL of the reaction mixture was transferred to each well of a MicroAmp Optical 96-Well Reaction Plate with Barcode (Applied Biosystems, cat# 4306737) on ice. The plate was sealed with MicoAmp Optical Adhesive Film (Applied Biosystems, cat# 4311971) and centrifuged at 1,200 × g for 2 min. qPCR was performed on an ABI 7300 Sequence Detection System with the following thermocycler program: 30 min at 50 °C, 10 min at 95 °C, 15 s at 95 °C and 1 min at 60 °C (40 cycles). Relative gene expression changes were calculated using the ΔΔ*CT* method. For each experiment, the CT values for each gene were normalized to the CT values of the gene *ptaA*. Graphs and data analysis were performed using the GraphPad Prism 6. All qPCR data were generated from three independent experiments. These experiments were performed using the same experimental protocol published by our lab in previous work [8, 17].

**Table:** 

Material	Amount / Reaction (20 μL)	Triplicate + Excess (88 μL)
SYBR	10 μL	44 μL
Primer (5 nM)_forward	1.5 μL	6.5 μL
Primer (5nM)_reverse	1.5 μL	6.5 μL
Rt enzyme	0.16 μL	0.704 μL
RNA	30 ng	132 ng
Water	20 μL	88 μL

### Statistical Methods

The GraphPad T test calculator was used to perform Student’s t-test throughout these studies (see link for details: https://www.graphpad.com/quickcalcs/ttest1/). Specifically, we performed the unpaired t-test to compare the means of two groups. The mean of each group being compared was determined independently, then compared using the Student’s t-test, and p-values were calculated relative to the vehicle (DMSO) control group.

### UV-visible spectroscopy to determine metal(II)-binding with RA-HQ-12

A mixture DMSO (970 μL) and RA-HQ-12 (30 μL of a 10 mM DMSO stock) was added to a 1.5 mL cuvette. In a separate cuvette, DMSO (955 μL), RA-HQ-12 (30 μL of a 10 mM DMSO stock), and metal(II) cation (15 μL of a 10 mM water solution) were added and thoroughly mixed. Spectral scans were then obtained from 300 to 700 nm in 10 nm increments at various time points (1 min, 5 min, 10 min, 30 min, 1 h, 20 h). The elevated absorbance between 380–480 nm for nickel(II)/cobalt(II) and 400–700 nm for iron(II) indicates the formation of RA-HQ-12:metal(II) complex formed in a 2:1 ratio. Experimental notes: Metal(II) salts used during these studies: NiSO_4_, CoSO_4_, Fe(NH_4_) 2(SO_4_)_2_·6H_2_O. The binding of RA-HQ-12 to Ni(II) and Co(II) was complete within 1 min, with no significant differences observed at later time points. Data were analyzed and plotted using GraphPad Prism 6.

## Supplementary Material

ESI file

**Supplementary information** The online version contains supplementary material available at https://doi.org/10.1007/s00044-025-03471-9.

## Data Availability

No datasets were generated or analysed during the current study.

## Abbreviations

ATCC: American Type Culture Collection
*crtM*: squalene desaturase (gene)
h: hour(s)
HQ: halogenated quinoline
*ilvC*: ketol-acid reductoisomerase (gene)
*isdB*: iron-regulated surface determinant B (gene)
MBEC: minimum biofilm eradication concentration
MIC: minimum inhibitory concentration
min: minute
MRSA: methicillin-resistant Staphylococcus aureus
*nasE*: assimilatory nitrite reductase (gene)
*opp1C*: peptide/nickel transport system permease protein (gene)
RT-qPCR: reverse transcription quantitative real time polymerase chain reaction
RA-HQ-12: analogue RA-HQ-12 (from the reductive amination HQ series)
*ribA*: riboflavin biosynthesis protein (gene)
*sbnC*: staphyloferrin B synthetase (gene)
*splB*: serine protease (gene)
μL: microliter
μM: micromolar
