# Supplementary material for: Halogenated quinoline kill agent rapidly induces iron starvation of Staphylococcal biofilms
Source: Med Chem Res. 2025 Sep 16;34(11):2309–17. Online ahead of print. doi: 10.1007/s00044-025-03471-9 (PMC12523839; doi:10.1007/s00044-025-03471-9)
Supplement: Supplementary file 1 — Supporting Information [file 44_2025_3471_MOESM1_ESM.pdf]

Supporting Information

Halogenated Quinoline Kill Agent Rapidly Induces Iron Starvation of *Staphylococcal* Biofilms

Robert W. Huigens III[a,b,c,d]*, Ke Liu[d], Nana Shao[a], Qiwen Gao[a]

[a]Department of Pharmaceutical and Biomedical Sciences, College of Pharmacy, University of Georgia, Athens, Georgia 30602, United States. [b]Department of Chemistry, Franklin College of Arts and Sciences, University of Georgia, Athens, Georgia 30602, United States. [c]Department of Infectious Diseases, College of Veterinary Medicine, University of Georgia, Athens, Georgia 30602, United States. [d]Department of Medicinal Chemistry, Center for Natural Products, Drug Discovery and Development (CNPD3), College of Pharmacy, University of Florida, Gainesville, Florida 32610, United States.

*Corresponding Author Robert.Huigens@uga.edu

Table of Contents

1.) Primers Used in This Study………………………………………………………………………………. S3

2.) RNA Quality Control……………………………………………………………………………………….. S4

3.) RT-qPCR Results………………………………………………………………………………………………… S7

1.) Primers Used in This Study.

a.) Primers used for MRSA 1707

Gene	Symbol	Forward Primer	Reverse Primer
MW1668	*ptaA*	TCCTAGCGAGTTCAGTTGCA	CCAATGGAATGTAGCTGCGA
MW1011	*isdB*	CCAGCAGCAAAAGCCACTAA	CGAGAGTTTGGTGCGCTATG
MW0091	*sbnC*	TCCGGTACATCCTTGGCAAT	GACATGGTTATACGGTGCGC
MW2389	*opp-1C*	TGTTTGGTATGGGTGCCGAA	CAGTAGGCGCTTTGACACCT
MW1709	*ribA*	ACACAGAAGCGGCTGTTGAT	TCTTGTCCTTTCGCCATCGT
MW1754	*splB*	TCAAGTTGAAGAGCGTGCAA	GGTGATCCAGAGTTTCCGCT
MW1980	*ilvC*	GCTTGCATTTGCTCATGGCT	ACCACCGCAAAGTACTGCTT
MW2483	*crtM*	AGAAAAGCGGTTTGGGCAAT	TGTGCAACATGCTGAAGTGC
MW2321	*nasE*	CTGAAGGGACAGTGAGTGGG	ACGTTCCCGTCGTAACTTCT

b.) Primers used for *S. epidermidis* 12228

Gene	Symbol	Forward Primer	Reverse Primer
SE0681	*ftsZ*	GTGGATTAGGTGCTGGTGCT	GCGCTTACGACCTTCGAAAC
SE1770	n.a.	AAGGGCATCCAACACATCCT	GAAGTGGCTCGAGGTAGCAT
SE2114	*feoB*	CGCTGGTGTGGGTTCTGTAT	CGGTAAGCGTGCTGAACATG
SE0516	n.a.	GCATCGATAGGAACGTCCGT	ACAACAGTTGCTTGTTCACCT
SE1439	n.a.	GGCTAAAGAGGGTCGTGGTC	TGGAAATAAGTGCCCCGGAC
SE1062	n.a.	TCACGCCCTGCTTGTAACAT	CTCGCGTGACAAGAGGGATT
SE1974	n.a.	GGGCCAAACTGGGAAGATGA	CCAACCTCGACAGGCTTCTT

2.) RNA Quality Control.

RNA concentration was determined on Qubit® 2.0 Fluorometer (ThermoFisher/Invitrogen, Grand Island, NY), RNA quality was assessed using the Agilent 2100 Bioanalyzer (Agilent Technologies, Inc.). Total RNA for these experiments was 53 - 723 ng/µL with RNA integrity numbers (RIN) = 9.1 - 6.8. Quality control data for each independent experiment is presented below and obtained from the Interdisciplinary Center for Biotechnology Research (ICBR) at the University of Florida.

a.) MRSA 1707 biofilms treated with RA-HQ-12 at 1 µM for 4 hours

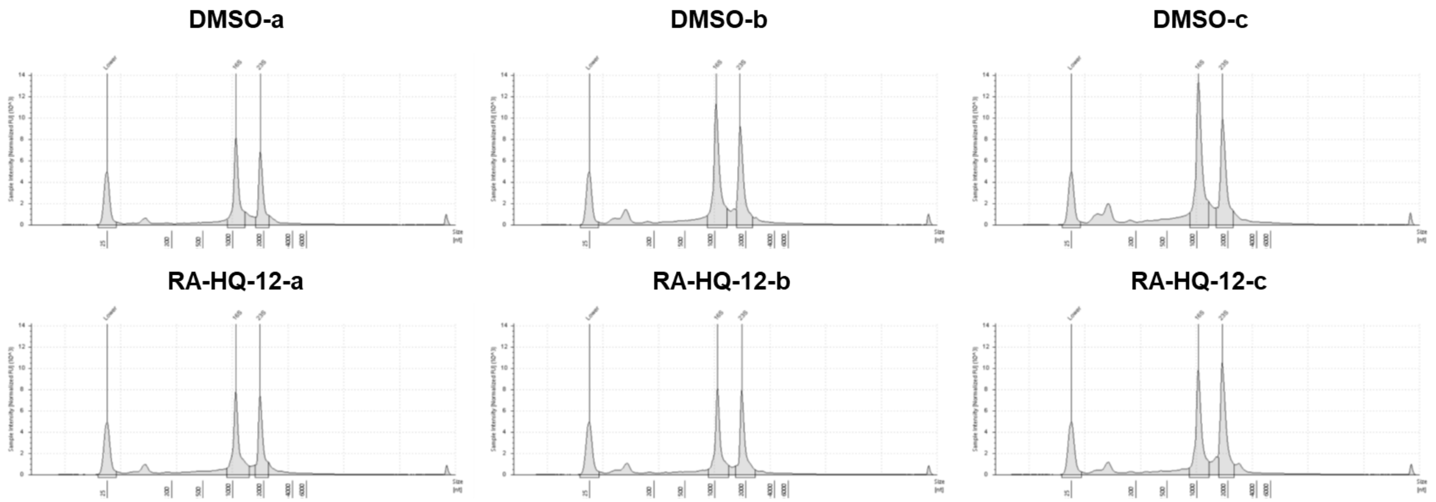

Sample	[ng / µL]	RIN
DMSO-a	389.2	8.6
DMSO-b	406.7	8.3
DMSO-c	366.6	8.6
RA-HQ-12-a	217.9	8.6
RA-HQ-12-b	345.8	8.3
RA-HQ-12-c	318.1	8.4

b.) MRSA 1707 biofilms treated with RA-HQ-12 at 1 µM for 2 hours

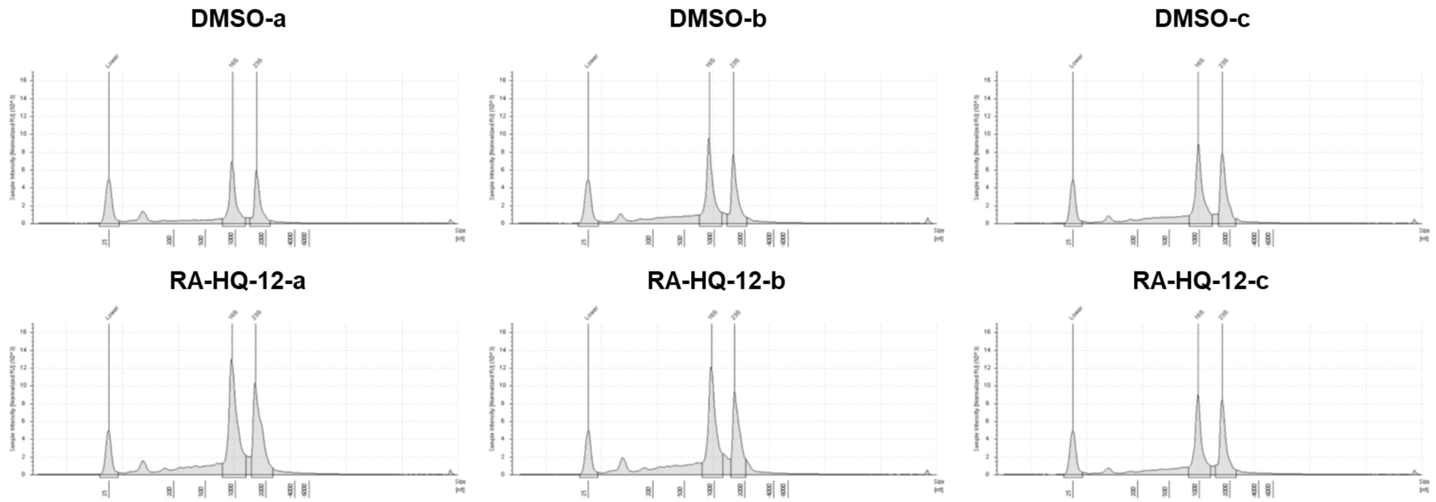

Sample	[ng / µL]	RIN
DMSO-a	137.8	8.8
DMSO-b	178.5	8.4
DMSO-c	213.3	8.5
RA-HQ-12-a	483.5	8.5
RA-HQ-12-b	384.4	8.3
RA-HQ-12-c	210.8	8.6

c.) MRSA 1707 biofilms treated with RA-HQ-12 at 1 µM for 8 hours

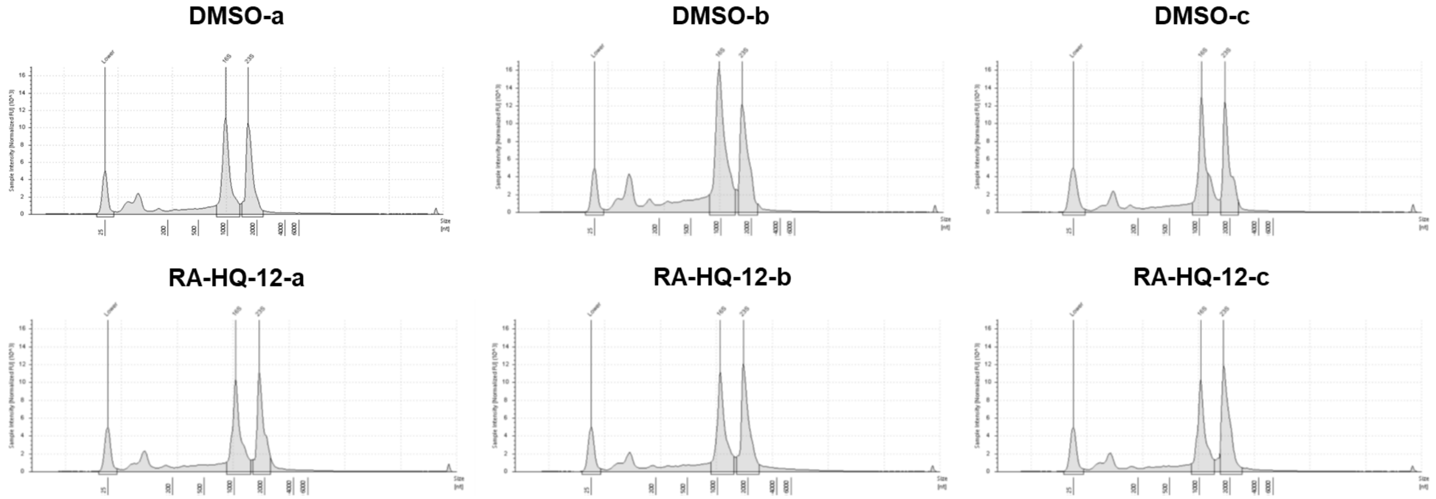

Sample	[ng / µL]	RIN
DMSO-a	413.0	8.7
DMSO-b	427.6	8.3
DMSO-c	649.6	8.8
RA-HQ-12-a	605.5	8.6
RA-HQ-12-b	723.6	8.7
RA-HQ-12-c	652.0	8.8

d.) MRSA 1707 biofilms treated with RA-HQ-12 at 1 μM for 20 hours

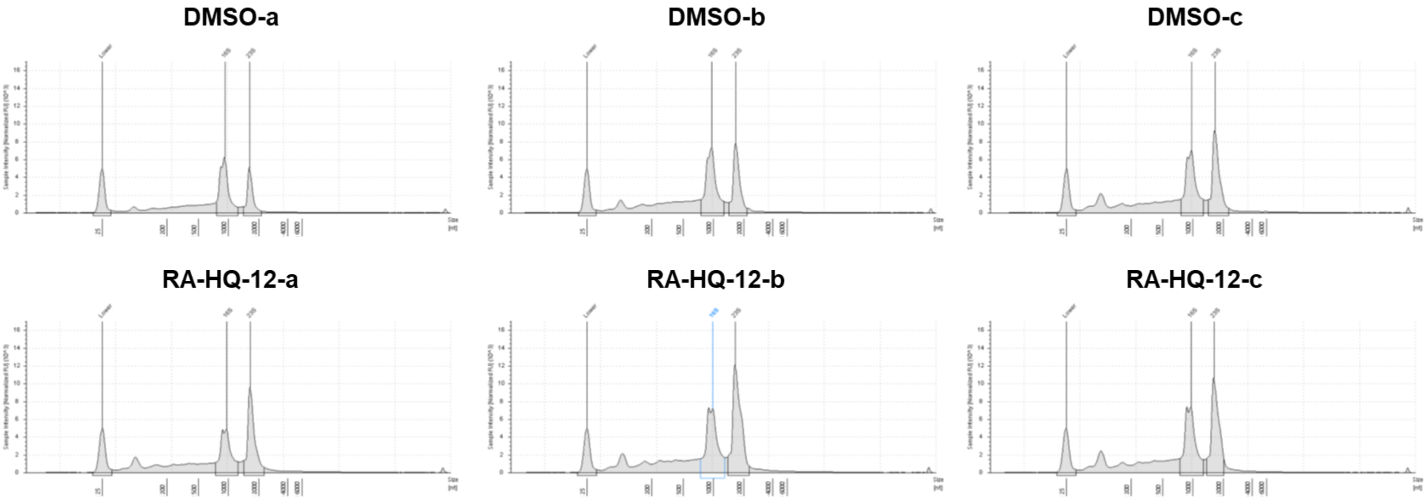

Sample	[ng / μL]	RIN
DMSO-a	226.1	7.5
DMSO-b	311.3	7.1
DMSO-c	384.3	7.0
RA-HQ-12-a	283.7	6.8
RA-HQ-12-b	421.7	6.9
RA-HQ-12-c	394.6	7.0

e.) *S. epidermidis* 12228 biofilms treated with RA-HQ-12 at 1 μM for 4 hours

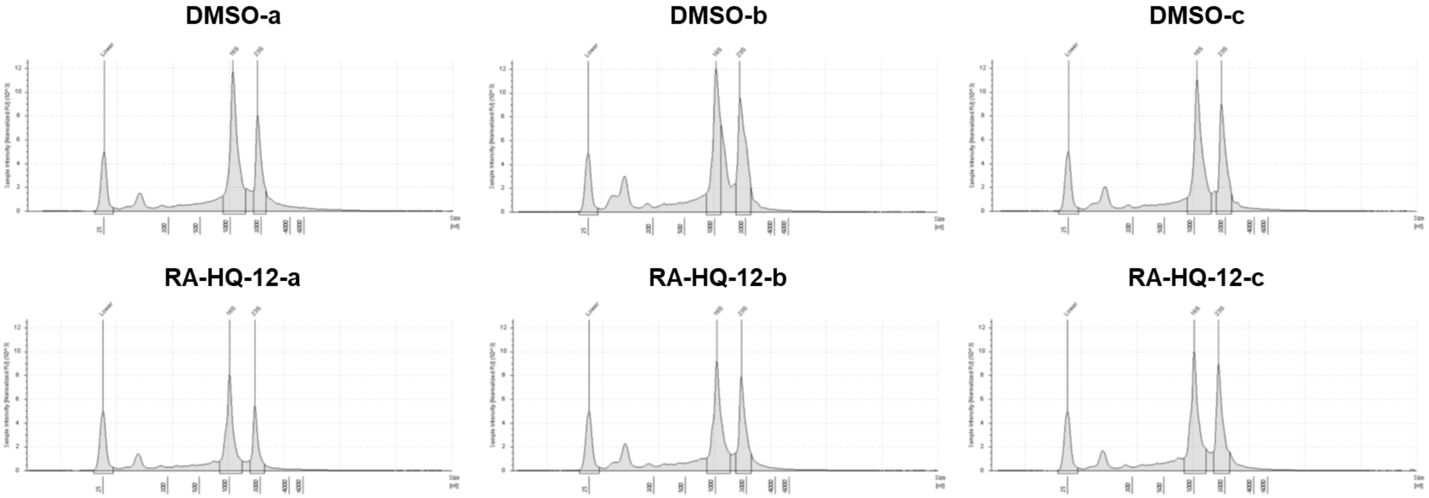

Sample	[ng / μL]	RIN
DMSO-a	363.3	9.1
DMSO-b	298.9	8.9
DMSO-c	457.7	9.0
RA-HQ-12-a	371.0	8.9
RA-HQ-12-b	98.3	9.1
RA-HQ-12-c	53.6	8.8

3.) RT-qPCR Results.

a.) MRSA 1707 biofilms treated with RA-HQ-12 at 1 μM for 4 h

Gene	DMSO	RA-HQ-12	P-value	Gene Information / Function
	Mean ± SD	Mean ± SD		
isdB	1.0 ± 0.01	3.2 ± 0.9	0.0276	iron-regulated surface determinant, mediates heme uptake
sbnC	1.0 ± 0.01	27.7 ± 8.3	0.0106	staphyloferrin B (siderophore) synthetase enzyme
opp-1C	1.0 ± 0.02	5.2 ± 2.1	0.0464	peptide/nickel transport system permease protein
ribA	1.1 ± 0.02	3.8 ± 0.6	0.0022	riboflavin biosynthesis protein
splB	1.1 ± 0.06	3.8 ± 1.3	0.0471	serine protease SplB
ilvC	1.0 ± 0.01	1.1 ± 0.4	0.9291	ketol-acid reductoisomerase, biosynthesis of branched-chain amino acids
crtM	1.1 ± 0.01	0.25 ± 0.2	0.0049	squalene desaturase, functions to synthesize staphyloxanthin (virulence)
nasE	1.0 ± 0.03	0.02 ± 0.01	<0.0001	assimilatory nitrite reductase, functions in nitrate assimilation

b.) MRSA 1707 biofilms treated with RA-HQ-12 at 1 μM for 2 h

Gene	DMSO	RA-HQ-12	P-value	Gene Information / Function
	Mean ± SD	Mean ± SD		
isdB	1.0 ± 0.00	8.1 ± 0.7	0.0001	iron-regulated surface determinant, mediates heme uptake
sbnC	1.1 ± 0.03	8.7 ± 2.7	<0.0001	staphyloferrin B (siderophore) synthetase enzyme
opp-1C	1.0 ± 0.01	12.7 ± 3.1	0.0057	peptide/nickel transport system permease protein
ribA	1.0 ± 0.02	2.5 ± 0.3	0.0015	riboflavin biosynthesis protein
crtM	1.0 ± 0.01	0.85 ± 0.04	0.0051	squalene desaturase, functions to synthesize staphyloxanthin (virulence)
nasE	1.0 ± 0.03	0.82 ± 0.18	0.1835	assimilatory nitrite reductase, functions in nitrate assimilation

c.) MRSA 1707 biofilms treated with RA-HQ-12 at 1 μM for 8 h

Gene	DMSO	RA-HQ-12	P-value	Gene Information / Function
	Mean ± SD	Mean ± SD		
isdB	1.0 ± 0.01	3.0 ± 1.6	0.0027	iron-regulated surface determinant, mediates heme uptake
sbnC	1.0 ± 0.02	28.4 ± 4.3	0.0008	staphyloferrin B (siderophore) synthetase enzyme
opp-1C	1.0 ± 0.02	14.2 ± 3.2	0.0045	peptide/nickel transport system permease protein
ribA	1.0 ± 0.02	1.2 ± 0.3	0.1910	riboflavin biosynthesis protein
crtM	1.0 ± 0.02	0.35 ± 0.1	0.0007	squalene desaturase, functions to synthesize staphyloxanthin (virulence)
nasE	1.0 ± 0.02	0.13 ± 0.04	<0.0007	assimilatory nitrite reductase, functions in nitrate assimilation

d.) MRSA 1707 biofilms treated with RA-HQ-12 at 1 μM for 20 h

Gene	DMSO	RA-HQ-12	P-value	Gene Information / Function
	Mean ± SD	Mean ± SD		
isdB	1.0 ± 0.01	1.8 ± 0.22	0.0054	iron-regulated surface determinant, mediates heme uptake
sbnC	1.0 ± 0.03	33.5 ± 0.6	<0.0001	staphyloferrin B (siderophore) synthetase enzyme
opp-1C	1.0 ± 0.03	2.9 ± 1.1	0.0034	peptide/nickel transport system permease protein
ribA	1.0 ± 0	3.0 ± 0.1	<0.0001	riboflavin biosynthesis protein
crtM	1.0 ± 0.01	0.21 ± 0.1	0.0002	squalene desaturase, functions to synthesize staphyloxanthin (virulence)
nasE	1.1 ± 0.04	0.82 ± 0.1	0.0355	assimilatory nitrite reductase, functions in nitrate assimilation

e.) ***S. epidermidis*** **12228 biofilms treated with RA-HQ-12 at 1 μM for 4 h**

Gene	DMSO	RA-HQ-12	P-value	Gene Information / Function
	Mean ± SD	Mean ± SD		
SE1770	1.1 ± 0.04	47.8 ± 45.9	0.0461	siderophore biosynthesis
SE2114	1.0 ± 0	12.8 ± 0.5	<0.0001	ferrous iron transport protein
SE0516	1.0 ± 0.01	13.1 ± 2.6	0.0014	ferrichrome ABC transporter
SE1439	1.0 ± 0.01	3.2 ± 1.1	0.0498	riboflavin biosynthesis
SE1062	1.0 ± 0.06	1.4 ± 1.3	0.5927	oligopeptide transporter
SE1974	1.1 ± 0.07	1.4 ± 0.4	0.3578	nitrate reductase
